# Identification of cervical epidural space: A comparison study between contrast spread and loss of resistance techniques

**DOI:** 10.3389/fpain.2022.1000209

**Published:** 2022-12-20

**Authors:** Yakov Perper

**Affiliations:** Department of Anesthesiology, Mount Sinai Queens, New York City, NY, United States

**Keywords:** fluoroscopy only method, cervical epidural steroid injection, contrast spread technique, loss of resistance technique, epidural space identification

## Abstract

**Background and objectives:**

Early epidural space identification is critical to the efficacy and safety of cervical epidural steroid injections (CESI). Currently, the accepted method for epidural space recognition is the loss of resistance technique (LORT). I hypothesized that the contrast spread technique might recognize epidural space concurrently with or sooner than LORT and that smaller needles might be employed with the fluoroscopy only method but not with LORT. To test my hypotheses, I conducted a comparison study.

**Methods:**

The study participants were patients from my practice with a clinical diagnosis of cervical radiculitis divided into two groups of 20 each, who underwent CESI with either an 18 G or a 25 G Tuohy needle. All CESIs were performed utilizing the fluoroscopy only method. Then, LOR was tested using an Epidrum device that was observed for 30 s; if the Epidrum deflated, the result was positive.

**Results:**

LOR was positive in 12 out of 20 patients in the 18 G group and 2 out of 20 in the 25 G group. The 95% confidence interval test for proportion revealed a statistically significantly lower rate for epidural space detection by LORT in both groups: [0.385, 0.815] in the 18 G and [-0.031, 0.231] in the 25 G group. Statistical significance of the difference between groups in LOR accuracy rate (60% vs. 10%) was confirmed by *z*-test for independent proportions: *z* = 3.31 (*p* < 0.001), Cohen's *h* = 1.13.

**Conclusion:**

Fluoroscopy only method might be a safer alternative to LORT as it employs a different concept, might recognize epidural space sooner, and favors smaller needles.

**Clinical trial registration:**

NCT05260294.

## Introduction

Early epidural space identification is critical to the efficacy and safety of cervical epidural steroid injections (1). It is paramount in the prevention of serious complications such as postdural puncture headache (PDPH) and spinal cord damage. Currently, loss of resistance technique (LORT) combined with fluoroscopic guidance is the preferred practice employed for cervical epidural steroid injections in the United States (2). Fluoroscopy is recommended for all cervical interlaminar injections to ensure correct depth of needle insertion and to avoid spinal cord penetration (3), and is obligatory (two appropriate planes are required) for the confirmation of the proper epidural spread after LOR is achieved (4). Although predominantly safe, LORT, even with fluoroscopy, carries a risk of spinal cord penetration and injury when performed at the cervical spine due to objective reasons (5). The “Fluoroscopy only” (2) method (FOM) might significantly diminish this risk as it eliminates the need for the LOR for epidural space recognition. With FOM, needle navigation and epidural space identification are performed under continuous or intermittent fluoroscopic guidance. Contralateral oblique (6, 7) and anterior-posterior fluoroscopy are employed for needle advancement from the skin toward the epidural space, and the contrast spread technique (CST) (8, 9) is used for epidural space identification. Despite the apparent theoretical and practical advantages of FOM over LORT in cervical epidural injections, there was no published research comparing the two techniques. For this reason, I decided to perform a study where the following two hypotheses were tested:

a) Contrast spread technique may recognize the epidural space concurrently with or sooner than LORT;
b) LOR is significantly less reliable with a 25 G Tuohy needle compared to an 18 G Tuohy needle.

## Methods

The study participants were patients of Astoria Pain Management, New York, United States (age 28–72 years) with a clinical diagnosis of cervical radiculitis. The Canadian SHIELD Ethics Review Board approved this study (July 18, 2019. REB tracking number: 19–06–002), conducted from August 19, 2019, to October 8, 2019. There was no funding for this study. Patients were eligible for the study if they met the criteria for cervical ESI, which included clinical and recent MRI findings confirming the diagnosis of cervical radiculitis and inadequate pain relief with conservative care for more than 3 months. Other criteria were if the procedure was covered by medical insurance, and if they signed informed consent. Patients were excluded from the study if they were taking anticoagulants or had serious comorbidities such as congestive heart failure or uncontrolled diabetes. Fifty-four patients underwent cervical ESI during the research period; a total of 45 patients agreed to participate, and 40 completed it. Among the patients who dropped out of the study, three had vascular uptake and the procedure was aborted, one refused to continue with the injection due to pain and anxiety (we did not provide sedation for cervical ESIs in the study), and on one occasion, a 20 G Tuohy needle was employed in error.

The patients were divided into two groups of 20 each and underwent CESI with either an 18 G or a 25 G Tuohy needle. Patient selection for the 18 G and 25 G groups was not random. For example, the preference was given for a 25 G needle when the procedure was performed at the C5–6 level (25 G/18 G = 15/4) and for an 18 G (18 G/25 G = 16/5) when the needle was inserted at C6–7 or C7-T1 level (Table 1) as an epidural space at the C5–6 spinal segment is narrower compared to the lower levels. The smaller needles were also favored for females (25 G/(25 G + 18 G) = 17/24) vs. males (25 G/(25 G + 18 G) = 3/16) (Table 1) because the average female neck is smaller, and the cervical spinal spaces are lesser compared to the male neck (10).

**Table 1:** 18 G and 25 G needles selection between sex and cervical levels.

	Male	Female	C5-6	C6-7	C7-T1
18 G	13	7	4	14	2
25 G	3	17	15	5	0

**Figure 1:**
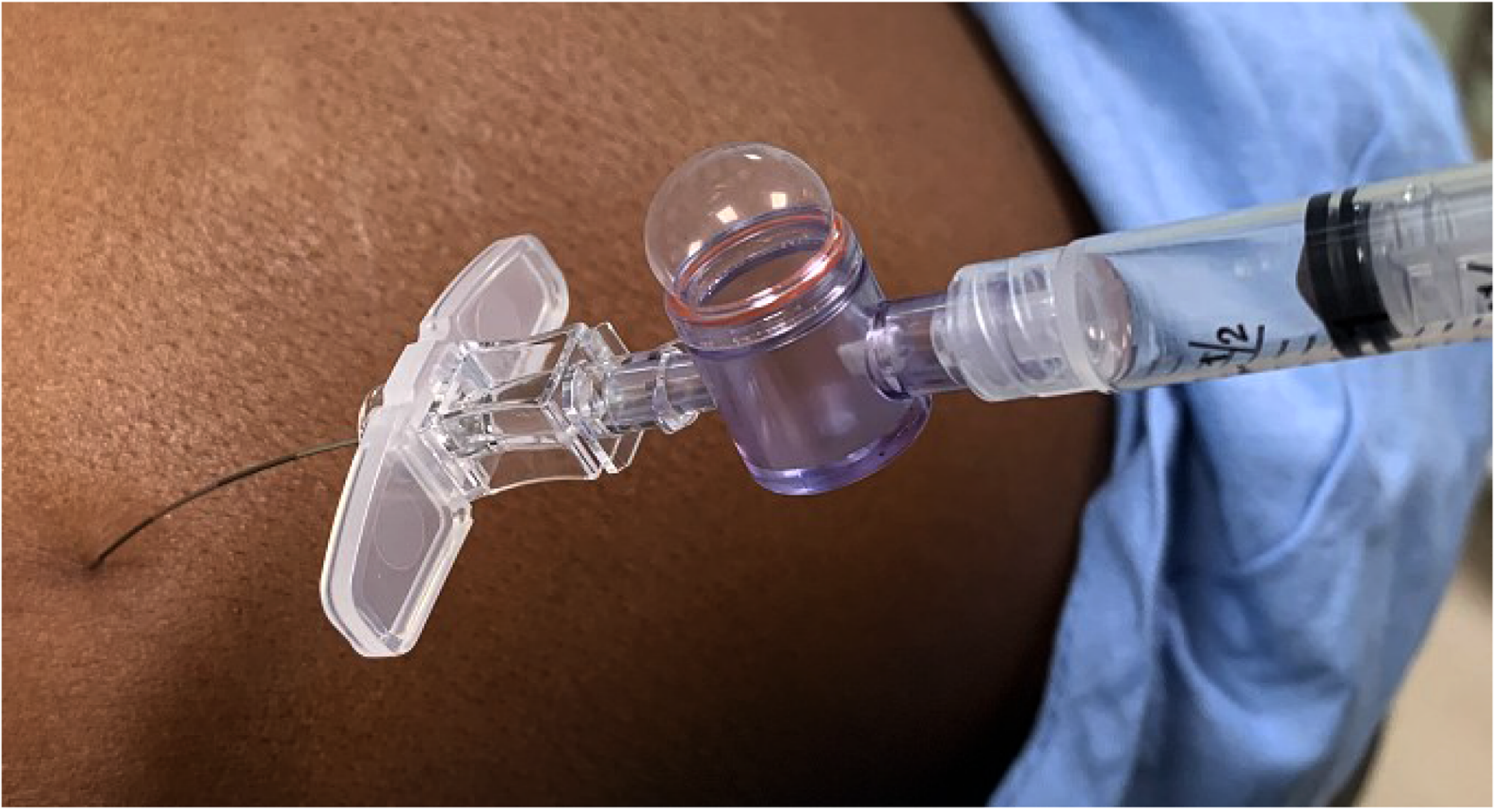
Epidrum. The Epidrum connected to a 25 G Tuohy needle is still inflated after 30 s. The result is negative.

The skin was anesthetized with 1% lidocaine in the 18 G group but not in the 25 G group. All CESIs were performed utilizing the FOM when the needle was navigated from the skin toward the epidural space paramedially under anterior-posterior (AP) and contralateral oblique fluoroscopy (CLOF), and the contrast spread technique was employed for epidural space identification (9). With the needle positioned at the ventral interlaminar line (VILL) (11), the investigator incrementally injected contrast, no more than 0.1 ml at a time, monitoring cautious needle advancement through the ligamentum flavum until the epidural spread appeared on the screen (12). After radiological confirmation of the epidural spread, LOR was tested using an Epidrum® device (Exmoor Innovations Ltd., Somerset, United Kingdom) (Figure 1). The Epidrum device was chosen as the author considered it an objective and reproducible method for epidural space identification independent of the provider's skill with the LORT, and because its success rate is comparable to the results attained by trained anesthesiologists employing the LOR syringe (13, 14, 15). Subsequently, accompanied by the radiology assistant, the investigator observed the Epidrum for 30 s or more; if the Epidrum deflated, the result was positive. However, if the device remained inflated, the result was reported as negative. The collected data was then analyzed.

## Results

Performing statistical analysis for the study results was a challenging process. There were three investigations:

1) Within group 1; 18 G (LORT vs. CST),
2) Within group 2; 25 G (LORT vs. CST), and
3) Between groups 1 and 2 (LORT 1 vs. LORT 2).

The *t*-test could not be employed to test the hypothesis for any of the three experiments because the results were presented as proportions, not means. Additionally, the Chi-squared or *Z*-tests for LORT vs. CST within groups 1 or 2 could not be used because the CST detection rate was 100% in both groups (i.e., the failure rate was 0). Therefore, the Confidence Interval Test for a Proportion was employed to estimate the confidence interval (CI) between LORT and CST within groups 1 and 2. The 95% CI was [0.385, 0.815] for group 1 (LORT 60%; 18 G) and [-0.031, 0.231] for group 2 (LORT 10%; 25 G), confirming that LORT had a statistically significantly lower rate of epidural space detection than CST within each group (Table 2).

**Table 2:** The 95% confidence interval test for proportions of epidural space detection by LORT within 18 G and 25 G groups.

	x	n	*p*	Margin of error for 95% CI	Lower Limit	Upper Limit
LORT 18 G	12	20	60	0.215	0.385	0.815
LORT 25 G	2	20	10	0.131	−0.031	0.231

The rate of epidural space detection by CST in both groups is 100%. The 95% confidence interval test for proportions confirmed that epidural space detection by LORT is significantly lower compared to the CST. x-positive results, n-number of patients, *p*-percentage of positive results.

The *Z*-test for Independent Proportions was utilized to compare the epidural space detection between LORT 1 (group 1; 18 G) and LORT 2 (group 2; 25 G). Since CST detected 100%, it was employed as a benchmark to conduct a hypothesis test of proportion, using H_0_: *p* = 1; Ha: *p* < 1. There was a statistically significant difference between the proportions of epidural space detection by LORT with 18 G compared to 25 G needles: *z* = 3.31, *p* < 0.001, Cohen's *h* = 1.13 (Table 3).

**Table 3:** The *z*-test for independent proportions for the difference in the epidural space detection by LORT between 18 G and 25 G groups.

		x	n	*p*
LORT 18 G		12	20	60
LORT 25 G		2	20	10
Significance level	Pooled proportion	z	*p*-value	Effect size Cohen's h
0.05	0.35	3.31	0.0009	1.13

CST, 20 (100%) was employed as a benchmark. Since *p*-value: 0.0009 < 0.05, H_0_ was rejected. As Cohen's h > 0.8, the difference in LORT detection rate between 18 G and 25 G needles is large. x–positive results, n–number of patients, *p*–percentage of positive results.

## Discussion

The study findings confirmed that CST could recognize epidural space concurrently with or sooner than LORT. This might be due to the fundamental difference between the two techniques utilized for epidural space identification. With CST, the force generated by the syringe plunger pushes the contrast matter inside the epidural space as soon as the needle tip enters it, as the density of the epidural space is significantly less than that of the ligamentum flavum, and the epidural space is potentially expandable (1). Similar mechanics are employed with LORT. However, with LORT, instead of visualization of the injected air or saline, the provider is testing a tactile sense of change in the resistance at the tip of the plunger. Therefore, to achieve LOR, the needle bevel has to be introduced inside the epidural space at a certain depth, which can be more than what is needed for the contrast, air, or saline to gain access inside it. As soon as the tip of the needle enters the epidural space, the injected contrast appears on the screen of the monitor as an epidurogram. This is why LOR may be recognized simultaneously with visualized contrast spread but never precedes it. However, the contrast spread might be identified before LOR no matter which needle is used.

The main objective of the LORT is the LOR. The purpose of CST is the contrast spread beyond the VILL. This could explain why CST might be safer. When the focus is on the loss of resistance, there is always a possibility of needle penetration beyond the dura, even in the hands of an expert. With attention to the contrast spread, this chance is much smaller, simply because there is no need for needle advancement with the beginning of the ventral (epidural) dye distribution. *In other words, while LORT can be false negative, CST, theoretically, cannot be.* Contrast injection through the needle positioned inside the epidural space can result in either an epidural spread, vascular opacification, or a combination of both. Moreover, the visual assessment of the image on the screen of the *C*-Arm monitor is more informative than the tactile sense at the tip of the LOR syringe.

It is empirically established that larger needles are more reliable with LORT. The majority of providers employ 18 G or 20 G epidural needles, fewer employ 22 G (2), and almost none 25 G. It was difficult to find 25 G Tuohy needles for the study. Nevertheless, it was surprising to see such a remarkable difference in the results between the 18 G and 25 G needle groups. This phenomenon could be explained by the effect of the perceived resistance with different gauge needles on the sensitivity of the LORT (16). The higher the resistance, the lower the sensitivity of the test. The resistance might be calculated with the Poiseuille equation, also known as Poiseuille law, a statement in physics that describes the relationship between the velocity of the steady flow of a fluid through a narrow tube (such as a blood vessel or a catheter) and the applied pressure, tube radius, tube length, and the coefficient of viscosity (17).

**Figure 2:**
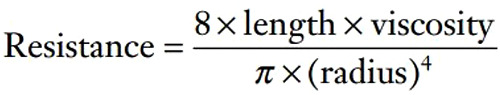
Poiseuille's equation.

Poiseuille's law can be successfully applied to air/fluid flow through the epidural/spinal needle. Based on the equation, the resistance is inversely related to the fourth power of the radius (Figure 2). The inner diameter of the 18 G Tuohy needle is twice and half that of 25 G: ≈1 mm vs.≈0.4 mm. Thus the resistance sensed by the physician performing the procedure with LORT using a 25 G needle is ≈40 times higher than if he would have performed the same injection with an 18 G needle.

Although more challenging in maneuverability, a smaller needle is less painful and can result in fewer complications. A larger needle predisposes patients to develop epidural hematomas (18) or PDPHs (19). A smaller needle has a shorter bevel and thus has to penetrate the epidural space less for epidural access. This may be advantageous in situations in which the epidural space is narrow, such as at the cervical spine. Also, it might permit needle placement at higher cervical levels that are inaccessible with traditional techniques employing larger needles. Thus, it might ease access to the targeted area of the patient's pathology eliminating the need for more complicated techniques, such as transforaminal or catheter-directed CESIs.

Both techniques may be combined when the physician draws contrast instead of air or saline, making it possible to test the tactile sense of LOR simultaneously with the visual assessment of the radiographic contrast spread (20). However, in such instances, the physician must be aware of altered resistance inside the LOR syringe and epidural needle as the viscosity of the contrast is much greater than that of air or saline.

I found FOM much less stressful than LORT. I attributed this to the higher degree of certainty with this technique, and the better knowledge of, and control over the needle tip positioning it provides. Since the summer of 2014, I have performed more than 2,300 cervical ESIs with FOM. The most frequent complication was accidental intravascular penetration observed during contrast injection. However, the number of vascular penetrations decreased dramatically after the replacement of the 25 G Quincke cutting-point needles (BD. Franklin Lakes, NJ, United States) with the 25 G AccuTarg curved tip short-bevel spinal needles (Hakko Co., Ltd. Chikuma-Shi, Nagano, Japan). There were few timely recognized subdural placements. There was not a single case of intrathecal spread or spinal cord needle penetration.

## Limitations and critique

One investigator, the lack of randomization, a small sample of patients, and a single location are the limitations of this study.

Contrast injection before LOR testing might render LOR less reliable. This is probably due to the increased resistance as the contrast is more viscous than traditionally employed air or saline. Needle flushing with normal saline before testing LOR might alleviate this problem.

FOM may increase fluoroscopy time as it employs fluoroscopy more than LORT. However, the significance of this increase is questionable as the neck's imaging requires less radiation, and a cumulative dose might not differ markedly. It will be interesting to see a study on this subject.

Some experts would not trust the efficacy of the Epidrum device in LOR (21). The author was looking for an objective way of epidural space identification independent of his skill in LOR and had chosen the Epidrum device to diminish bias. Perhaps, a better study design would be to investigate how often contrast enters the epidural space without perceived LOR assessed by the LOR syringe at or beyond VILL. Although the results can differ from the presented study, it would not alter the tendency. The detection rate of the epidural space by LOR will remain the same or lower compared to the contrast spread technique and the gap between the two techniques will increase with the reduction in the needle's inner diameter.

Also, one might critique the study for improper terminology. In the study, resistance is tested when the needle tip has been already positioned inside the epidural space. It is different from the traditional LORT when a physician is continuously or intermittently testing pressure at the syringe plunger while advancing a needle through dense tissues until the resistance changes/decreases, thus detecting the appearance of the epidural space. So, it is proper to state that the presence or absence of the low resistance, distinctive to the epidural space, rather than LOR, was tested in the study. However, this departure is not principal because improved sensitivity of the test would change the results but not the key findings of the study.

It is important to differentiate between the contrast spread technique and the fluoroscopy only method. While the term “CST” is employed solely for the process of epidural space identification (compared to the LOR), the term “FOM” is utilized for the procedure of needle depth recognition (visualization) from the skin toward the epidural space, with or without CST, during an epidural injection (compared to the LORT).

What is crucial, what distinguishes this study, is that:

a) Fluoroscopy only method is a novel technique, different from the traditional LORT, different from any other technique previously described in the literature on interlaminar epidural injections;
b) It employs a different concept that consists of the combination of fluoroscopic visualization and control over the needle navigation toward the epidural space utilizing AP and CLOF (on rare occasions, ipsilateral oblique fluoroscopy), and then, radiographic recognition (on the screen of the monitor) of the needle entrance inside it (CST). LORT, on the other hand, employs a tactile sense of resistance at the tips of the fingers and utilizes contrast injections for needle tip visualization if there is doubt regarding location (22), and for epidural space confirmation (4).

## Conclusion

In CST, the recognition of the needle entry into the epidural space can occur concurrently with or before LOR. 25 G epidural/spinal needles could be used safely with FOM but not with LORT as the sensitivity of the LOR decreases significantly with this needle size, rendering the technique dangerously risky. FOM might be a safer alternative to LORT as it employs a different concept, might recognize epidural space concurrently with or sooner, and because smaller needles pose less risk of complications. Further evidence is necessary to explore my findings.

## Data Availability

The original contributions presented in the study are included in the article/Supplementary materials, further inquiries can be directed to the corresponding author/s.
